# Reading characters in voices: Ratings of personality characteristics from voices predict proneness to auditory verbal hallucinations

**DOI:** 10.1371/journal.pone.0221127

**Published:** 2019-08-12

**Authors:** Kaja Julia Mitrenga, Ben Alderson-Day, Lucy May, Jamie Moffatt, Peter Moseley, Charles Fernyhough

**Affiliations:** 1 Department of Psychology, Durham University, Durham, England, United Kingdom; 2 School of Psychology and Clinical Language Science, University of Reading, Reading, England, United Kingdom; 3 School of Psychology, University of Sussex, Falmer, England, United Kingdom; 4 Department of Psychology, University of Central Lancashire, Preston, England, United Kingdom; Max Planck Institute for Human Cognitive and Brain Sciences, GERMANY

## Abstract

People rapidly make first impressions of others, often based on very little information–minimal exposure to faces or voices is sufficient for humans to make up their mind about personality of others. While there has been considerable research on voice personality perception, much less is known about its relevance to hallucination-proneness, despite auditory hallucinations being frequently perceived as personified social agents. The present paper reports two studies investigating the relation between voice personality perception and hallucination-proneness in non-clinical samples. A voice personality perception task was created, in which participants rated short voice recordings on four personality characteristics, relating to dimensions of the voice’s perceived Valence and Dominance. Hierarchical regression was used to assess contributions of Valence and Dominance voice personality ratings to hallucination-proneness scores, controlling for paranoia-proneness and vividness of mental imagery. Results from Study 1 suggested that high ratings of voices as dominant might be related to high hallucination-proneness; however, this relation seemed to be dependent on reported levels of paranoid thinking. In Study 2, we show that hallucination-proneness was associated with high ratings of voice dominance, and this was independent of paranoia and imagery abilities scores, both of which were found to be significant predictors of hallucination-proneness. Results from Study 2 suggest an interaction between gender of participants and the gender of the voice actor, where only ratings of own gender voices on Dominance characteristics are related to hallucination-proneness scores. These results are important for understanding the perception of characterful features of voices and its significance for psychopathology.

## Introduction

People form first impressions of others based on very limited behavioural information. Research on face perception has shown that people make automatic judgements about the trustworthiness of a novel face as rapidly as within 50–100ms [1, 2]. Similarly, recognition of facial expressions of emotions has been found to be a fast-acting process occurring between 23–28ms after looking at a face [3]. These automatic recognition processes are not reserved for the visual modality: listening to another person’s voice can be equally informative when making judgements about other people. Recent findings show that emotions are accurately recognised from nonverbal vocal cues, and the recognition can occur automatically between 300–360ms after exposure to a voice [4]. However, the time in which emotions are recognised from voices can vary–recognition of emotions might be different depending on the emotion type [5].

Voices are sometimes described as ‘auditory faces’, as they carry a wealth of information related to physical and personal characteristics of others [6]. Humans accurately estimate characteristics such as age, weight and height through listening to a voice alone [7, 8]. Krauss et al. [7] showed that people match vocal to facial identity in pictures with above 75% accuracy, and estimation of personal characteristics from voices is as accurate as when inspecting photographs. Detailed information such as waist-to-hip ratio and number of sexual partners have also been found to be accurately predicted from listening to a person’s voice [9].

Voices also carry cues to speaker’s personality characteristics, and people have been found to form consistent personality impressions when listening to strangers’ voices [10, 11]. Vocal attractiveness ratings have been observed to correlate with perceived traits of dominance, strength and assertiveness in male voices, while warmth, trustworthiness, honesty and kindness were associated with perceived attractiveness of female, but not male voices [11]. Similarly, McAleer et al. [10] found that after listening to brief recordings of the word *hello*, impressions of attractiveness in male speakers were more likely to be associated with ratings of strength, while female voice attractiveness was associated with trustworthiness and warmth personality traits. These impressions can be made following less than a second of exposure to an unfamiliar voice, and can be summarised in a two-dimensional ‘social voice space’, reflecting Valence and Dominance vectors for personality traits [10]. Consistency in first impressions of personality characteristics ratings has been found in a number of other studies (e.g. [12, 13]).

Belin et al. [6] proposed a model through which the perception of the human voice occurs in three separate processes: identification of voices as produced by humans, recognition and differentiation between voices, and recognition of vocal affective states [6]. Substantial evidence suggests that these processes happen automatically within milliseconds of exposure to a voice. For example, humans show over 99% accuracy in discrimination between familiar and unfamiliar voices, where only familiar voices prompt ERP-associated components that are evident at between 210–245ms post-exposure in fronto-central areas [14]. Similarly, discrimination between human and non-human sounds relates to differences in auditory cortical activation, where human speech triggers stronger responses than computer-generated tonal stimuli [15]. Efficiency in vocal emotion recognition also appears to be an automatic and accurate process, occurring between 300–500ms after exposure to a voice [4]. The processes associated with vocal emotion recognition were found to be accurate and uncompromised even under demanding distraction conditions [4].

While understanding of voice perception is important in itself, it is also of potentially great significance for psychopathology. Frameworks building up on evidence from normal voice perception and predictive processing–such as the Auditory Processing Stream framework–are useful tools in improving the understanding of AVH [16]. According to this framework, processing of vocal information related to a voice’s identity, affect and location takes place in two separate pathways in the brain (‘streams’), each being a distinct source of prediction. However, because of the separation of the two auditory networks, the vocal information can be disrupted, resulting in perceptual errors [16]. Understanding of processes associated with normal voice perception could provide clues as to how and why auditory verbal hallucinations (AVH) can be unpleasant and distressing experiences.

AVH are commonly associated with psychiatric disorders (e.g., schizophrenia) but also occur in healthy samples [17, 18]. Studies estimate that around 1.2% of the general population have frequent hallucinatory experiences, and 7.3% report life-time prevalence of AVH [19, 20]. Studying hallucination-proneness in non-clinical populations allows for the potential investigation of underlying neural and cognitive processes associated with hallucinatory experiences. The same processes have been proposed to underlie these experiences in both clinical and non-clinical populations [21]. It is also beneficial for avoiding confounding factors that might be associated with studying these experiences in clinical populations–for example effects of antipsychotic medication or higher prevalence of psychotic symptoms.

Relationships that individuals form with their AVH can resemble those with voices encountered in everyday life. The social content of the voice, and its perceived identity, agency and personality, may be a primary part of the voice-hearing experience [22]. Voices are often recurring and personified, with some voice-hearers reporting that they would miss their voices if they disappeared [23]. The identity and character associated with the voice is often persistent throughout a lifetime [24] and can affect the type of relationship that is formed between a voice-hearer and a voice, subsequently defining the nature of the voice-hearing experience. Beliefs in relation to perceived omnipotence and malevolence/benevolence can in some cases be more powerful than the content of the voice itself when predicting the relationship with AVH [25]. Perceived voice identity characteristics have been shown to correspond with affective and behavioural responses to voices: malevolence appears to trigger negative emotions, and positive emotions are associated with voices perceived as benevolent [26]. While the experience of hearing voices in clinical populations is often considered to be a distressing experience, it is much more rarely so in non-clinical populations [26]. Non-clinical voice-hearers often report the content of their voices to be neutral or positive, while patients are more likely to experience negative and distressing AVH. This is crucial for determining individual need for care, where people with positive content of voices often do not seek it [17]. Further research investigating the association between voice personality judgements and distressing voices will be important, to test whether such judgements might lead to increased distress in clinical populations.

The processes associated with voice perception have rarely been studied in populations with hallucinatory experiences. However, there is evidence to suggest that voice identity and affect recognition are atypical in schizophrenia: research on perceived voice identity showed that schizophrenia is associated with poorer performance on voice recognition [27, 28]. Discrimination between self-produced and external voices has also been found to be impaired in schizophrenia, with self-produced voices often being misattributed to others [29]. In terms of vocal emotions, schizophrenia patients show significantly less accuracy in recognising emotions from voice stimuli [30]. Similar patterns in recognition of vocal emotions have been found in non-clinical participants prone to experience AVH [31]. However, voice identity recognition in non-clinical voice-hearers seems to be unimpaired, contrary to what has been found in clinical groups. ([32], for a review see [30]). This appears to be problematic in view of traditional continuum models of psychosis, where similar underlying mechanisms for psychotic symptoms would be expected in sub-clinical and clinical dimensions [32]. It has been speculated that deficits in voice processing e.g., impaired vocal identity recognition, can manifest in more advanced clinical stages, where other symptoms–including delusions–begin to co-occur with hallucinatory experiences [32].

It is not clear how hallucinatory experiences relate to automatic processing of different voice characteristics, for example perceived personality features. To address this, the present study investigated the perception of personality features in relation to auditory hallucination-proneness in non-clinical samples. If vocal cues to socially important information like personality are a key to the experience of AVH, then we may expect hallucination-proneness to be related to judgements about personality of voices. To our knowledge, this aspect of voice perception has not been previously studied in this context.

It should also be considered that any relation between perceived personality characteristics and auditory hallucination-proneness could be a product of other confounds, for example paranoid thinking or mental imagery abilities. Delusions and paranoid thinking are symptoms commonly associated with schizophrenia and psychosis. Paranoia has been previously linked with tendencies to overestimate perceived threat, as well as more rapid and extreme responses associated with reasoning and jumping to conclusion biases [33–35]. Evidence suggests that paranoia is a significant factor in overattribution of emotional characteristics to neutral stimuli in facial perception research (e.g., [36, 37]). Thus, paranoid thinking could confound the ratings of personality characteristics by leading to more extreme interpretations of ambiguous voices in the current study. Additionally, individual differences in imagery abilities might be another confounding factor when assessing the link between the perception of personality characteristics and hallucination-proneness. It has been previously observed that more vivid imagery is linked to higher hallucination-proneness scores in non-clinical populations [38, 39], and has been proposed to be a trait marker in schizophrenia [40]. However, some studies suggest the opposite, where mental imagery was not found to be different in schizophrenia, high hallucination-proneness and non-clinical populations [41, 42]. It remains unexplored whether the link between mental imagery and hallucination-proneness might affect perception of voice personality characteristics. If hallucination-proneness is characterised by heightened imagery abilities, we might expect it to have some impact on the perception of voices.

To address this, the relation between hallucination-proneness, paranoid thinking, imagery abilities and voice personality characteristics ratings were explored in two samples of non-clinical participants. The research was conducted with non-clinical participants to avoid the confounds of testing with clinical populations, such as use of anti-psychotic medication, or high prevalence of other psychotic symptoms. We asked participants to listen to recordings of short word articulations (*hello*, *thank you*, *okay*, *sorry*), and rate them on four personality characteristics (trustworthiness, aggressiveness, confidence and warmth) relating to the two dimensional ‘social voice-space’ consisting of Dominance and Valence dimensions of personality [10]. McAleer et al. [10] showed that personality characteristics including trustworthiness and warmth constituted the reported dimension of Valence, where aggressiveness and confidence both corresponded to the dimension of Dominance [10]. We further investigated if gender of voices and participants can relate to the perception of personality features. This is important to explore considering perception of hallucinated voices–they are often perceived as male voices and highly dominant in nature, in both male and female patients [43]. It is not clear how gender might affect perception of normal voices in relation to hallucination-proneness in non-clinical participants.

We first explored whether Dominance and Valence personality ratings predicted hallucination-proneness. Secondly, we investigated whether paranoia could account for any relation between auditory hallucination-proneness and personality ratings. We further tested whether any differences in voice personality ratings related to individual differences in imagery abilities. Finally, we explored whether these relations were dependent on the gender of the speakers in the voice task and the gender of the participants.

## Study 1

### Method

#### Participants

The sample consisted of 94 participants (79 females, 14 males and 1 other), aged 18–38 (*M* = 20.29, *SD* = 3.13). The majority of participants were White British (87.2%) and right-handed (85.1%). All participants were recruited from a university setting through departmental participant pool advertisements. Ethical approval was given by the Department of Psychology Ethics Sub-committee at Durham University. Participants received course credit for their participation.

#### Measures

**Revised Launay–Slade Hallucination Scale (LSHS-R; [44, 45]):** The nine-item self-report scale included statements used by McCarthy-Jones and Fernyhough [44], adapted from Morrison et al. [45]. The scale includes five auditory and four visual hallucination statements. Responses are made on a five-point Likert scale, from ‘Never’ (1) to ‘Almost always’ (4). Only the five items relating to auditory modality were used in the analysis (previous studies have also included the analysis of auditory items only (e.g., [46]). The scale has been shown to have high internal reliability [38].

**Persecution and Deservedness Scale (PADS; [47]):** Paranoid thinking and perceived deservedness were measured with a 10-item self-report scale. Paranoia answers are made on a five-point Likert scale, where answers range from 0 (‘Certainly false’) to 4 (‘Certainly true’). Deservedness answers are given following positive endorsement of ratings higher than two in the Persecution Subscale, and can range from 0 (‘Not at all’) to 4 (‘Very much’). For the purpose of the present study, only paranoia scores were included in the analysis (this includes items 1, 3, 5, 7, 9, 11, 12, 13, 15, 17 and 19).

#### The Plymouth Sensory Imagery Questionnaire (Psi-Q; [48])

Fifteen items from a 35-item scale were used to assess vividness of imagery in auditory, visual and emotional modalities. Ratings are given on a 10-point scale ranging from 0 (‘No image at all’) to 10 (‘Image as clear and vivid as real life’). Rating could range from 0 to 15.

#### Stimuli

Eighty short voice recordings (mean duration = 670ms) including articulation of four words (*hello*, *thank you*, *sorry* and *okay*) were created. In total, 20 different speakers were used to produce the stimuli (10 male and 10 female speakers). Each speaker was recorded saying each of the four words. The average age of the speaker was 20.75 years old, and the majority of them came from the South East or Midlands area of England, UK. The recordings were created with Zoom H2n Portable voice recorder. The recordings were edited for length and normalised for volume in Pydub (https://github.com/jiaaro/pydub).

#### Voice Personality task

**Fig 1:**
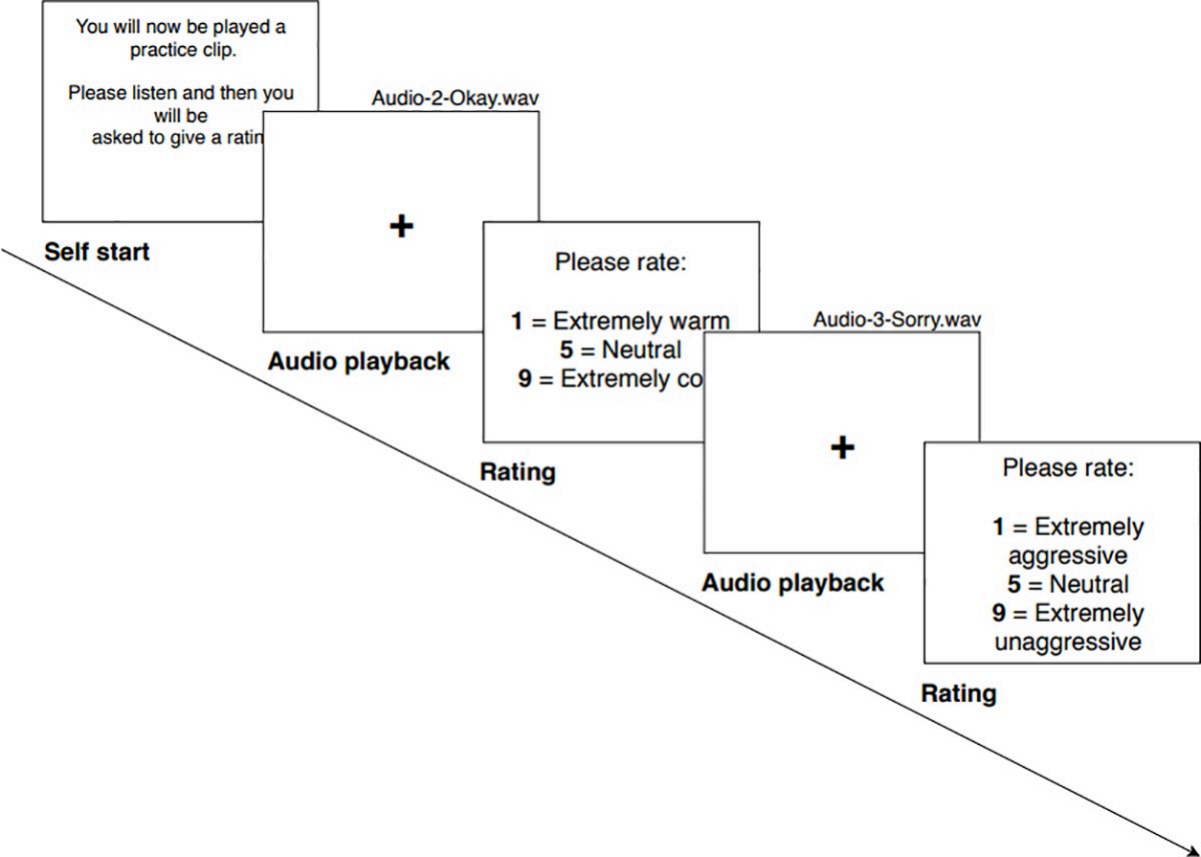
Example of experimental trials in the voice-personality task.

Participants rated each recording on a single personality characteristic only (aggressiveness, confidence, warmth or trustworthiness). Each participant rated eighty voice recordings, of which twenty were rated on confidence personality characteristics; twenty on aggressiveness; twenty on warmth; and twenty on trustworthiness. Therefore, each voice was rated on all personality characteristics throughout the task (where four words recorded for each speaker were rated on four personality characteristics). Ratings of the four words were counterbalanced for the four personality characteristics. The presentation order of voice recordings was randomised, and each voice received ratings on four of these personality characteristics. The ratings were made on a scale ranging from 1 (‘Extremely [characteristics]’) to 9 (‘Extremely un[characteristic]’). Lower scores indicated greater endorsement of personality traits. Participants gave their ratings by clicking a button displayed on a computer screen or pressing a corresponding key on the keyboard. Please see Fig 1 for the example of experimental trials.

#### Procedure

All data collection was carried out online. The Voice Personality task and questionnaires were presented online through the Testable platform. Participants were instructed to use headphones and carry out the experiment in a quiet environment. They were instructed to set their computer volume to comfortable listening level. Participants completed the Voice Personality task in the beginning of the online study, followed by the RLSHS-A, PADS and Psi-Q questionnaires. Participants were informed that we are not interested in any experiences that might have occurred whilst under the influence of drugs, at the start of the first questionnaire.

#### Data analysis

Data analysis was carried out in SPSS 20. Participant ratings for Trustworthiness and Warmth personality dimensions were summed to create a Valence variable, and Aggressiveness and Confidence ratings were summed to create a Dominance variable. The assumption of normality was not met for RLSHS-A and Psi-Q following inspection of normality tests, QQ plots, skew and kurtosis scores. Accordingly, natural logarithm transformation was applied to the RLSHS-A scores. Due to the data being negatively skewed, square root transformation was used to transform the Psi-Q scores. Non-transformed scores are reported in pairwise correlations. Relations between the Dominance and Valence voice personality ratings, RLSHS-A and Persecution were assessed with hierarchical regression analysis.

### Results

#### Relations between auditory hallucination-proneness (RLSHS-A), Valence and Dominance voice personality ratings, and paranoid thinking

Table 1 shows the correlation matrix for auditory hallucination-proneness scores (RLSHS-A), Valence and Dominance voice personality ratings and paranoid thinking (PADS). Neither Dominance (*p* = .06) nor Valence (*p* = .53) voice personality ratings correlated with auditory hallucination-proneness. Hallucination-proneness (RLHS-A) positively correlated with paranoid thinking (*p* < .001). Imagery abilities negatively correlated with Valence personality ratings (*p =* .006) and paranoid thinking (*p =* .017).

**Table 1:** Correlations between RLSHS-A, Valence and Dominance voice personality scores, paranoid thinking (PADS) and imagery (Psi-Q).

	Valence	Dominance	Paranoid Thinking	Imagery
RLSHS-A	.07	-.19	.39**	.03
Valence	-	.002	.16	-.29**
Dominance	-	-	-.13	.13
Paranoid Thinking	-	-	-	-.25*

*p<0.05

**p<0.001, two-tailed

#### Predicting hallucination-proneness (RLSHS-A) controlling for paranoid thinking and imagery abilities

A hierarchical regression analysis was performed to assess contributions of Dominance and Valence voice personality ratings, paranoid thinking (PADS) and imagery abilities (Psi-Q) in predicting hallucination-proneness (RLSHS-A). Valence and Dominance voice personality ratings were entered in the first block, followed by paranoid thinking (PADS) and imagery abilities scores (Psi-Q) in the second block, with auditory hallucination-proneness (RLSHS-A) as a dependent variable. Measures of multicollinearity were in the acceptable range (VIF<5). The results show that only Block 2 (*F*(4, 85) = 5.02, *p* = .001) significantly predicted auditory hallucination-proneness. In Block 1 (*R*^*2*^ *=* .05), only Dominance voice ratings significantly predicted auditory hallucination-proneness (β = -.22, *p* = .04). The addition of paranoid thinking and imagery ability scores in Block 2 significantly contributed to the model (*R*^*2*^ *=* .19; Δ *R*^*2*^ = .14, Δ *F*(2,85) = 7.55, *p* = .001), where paranoid thinking, β = .39, *p* < .001, significantly predicted auditory hallucination-proneness (RLSHS-A). The results are displayed in Table 2.

**Table 2:** Hierarchical regressions for predicting auditory hallucination-proneness in Study 1 and Study 2.

	Study 1				Study 2			
	Block 1		Block 2		Block 1		Block 2	
	B	*β*	B	*β*	B	*β*	B	*β*
Dominance	-.002	-.22*	-.001	-.19	-.06	-.40*	-.06	-.39**
Valence	0.00	.02	0.00	.003	.38	.19	.37	.19
PADS			.005	.39**			.01	.54**
Psi-Q			-.34	-.16			.02	.21*
*R*^*2*^	.05		.19		0.10		.44	
*F*	2.17		5.02		4.10		14.30	
**Δ** *R*^*2*^			.14				.34	
**Δ***F*			7.55				22.21	

* p < 0.05

**p < 0.001

#### Predicting Valence and Dominance ratings controlling for voice gender

We then investigated whether the relation between hallucination-proneness scores and ratings of male and female voices on Valence and Dominance personality dimensions were affected by the gender of participants. Previous research suggests a difference in the perception of male and female voices, where judged attractiveness is associated with different characteristics for both genders [10].

A multiple hierarchical regression was run, with Gender (of participants) entered in Block 1, followed by Valence and Dominance ratings for female and male voices (i.e., voice actors’ gender) in Block 2, with auditory hallucination-proneness (RLSHS-A) as the outcome variable. The gender of participants and ratings for different gender voices did not predict the scores for auditory hallucination-proneness (*p* > .05).

#### Summary

Study 1 investigated the relations between auditory hallucination-proneness, voice personality ratings in Valence and Dominance dimensions, paranoid thinking and imagery abilities. The results suggest that dominant personality judgements might be related to hallucination-proneness, but it is not evident that this is separate from paranoia/general delusional thinking. The results showed a significant relation between Dominance ratings of voices and hallucination-proneness; however, this relationship was no longer significant after the inclusion of paranoid thinking scores (PADS). Paranoia was found to be a significant predictor of hallucination-proneness in Block 2. There were no apparent gender effects in the relation of voice personality ratings and hallucination-proneness; however, there was a largely unequal gender split in the sample (84% females). In Study 2 we replicated the study in an online sample, aiming to recruit a sample with greater gender and age variability.

## Study 2

### Method

#### Participants

102 participants (41 females and 61 males), aged 18–60 (*M* = 32.02, *SD* = 10.06) took part in the study. The majority of participants identified as White (80.0%) and were right-handed (92.2%). Over half of responses (51.9%) came from English-speaking countries (see Table 3 for more demographic information). Participants were pre-screened for being native English speakers. All participants were recruited via the Prolific platform and received approximately £2.00 in reward for their participation. The study was designed in JsPsych (de Leeuw, J.R., 2015) and hosted on Durham University servers. The study was designed in a different platform to allow for a more representative population sample to be recruited from the Prolific platform (as we were not able to advertise the study there when it was hosted on the online platform used in Study 1). The full script for this experiment is available in the Open Science Framework at the address: doi: 10.17605/OSF.IO/ZXSQC. Ethical approval was given by the Department of Psychology Ethics Sub-committee at Durham University.

**Table 3:** Basic demographics (*N* = 102).

	Frequency	%
**Gender**		
Male	61	59.8
Female	41	40.1
**Country** *(top 5 listed)*		
United Kingdom	35	31.3
United States of America	15	14.7
Portugal	6	5.8
Spain	4	3.9
Ireland	2	1.9
**Ethnicity**		
White	82	80.4
Hispanic/Latino	11	10.8
Black/African American	4	3.9
Native American/American Indian	4	3.9
Asian/Pacific Islander	1	1

#### Measures

The same measures were used as in Study 1, including the Voice Personality task, hallucination-proneness questionnaire (RLSHS-A), paranoid thinking (PADS) and imagery abilities questionnaire (Psi-Q). Attention checks were used throughout the questionnaires to monitor participants’ engagement. In total four items were used and included instruction for participants to leave a question unanswered. Participants were not included in the study if they failed more than 50% of the attention checks. The study followed the same procedure as Study 1.

#### Data analysis

Data analysis was carried out in SPSS 20. Participant ratings for trustworthiness and warmth personality dimensions were summed to create a Valence variable, and aggressiveness and confidence ratings were summed to create a Dominance variable. The assumption of normality was not met for RLSHS-A, Valence and Dominance voice personality scores, paranoid thinking (PADS) and imagery abilities (Psi-Q), following inspection of normality tests, QQ plots, skew and kurtosis scores. Auditory hallucination-proneness scores (RLSHS-A), Valence voice ratings and imagery abilities scores were transformed using natural logarithmic transformation. Square root transformation was applied to negatively skewed distributions in Psi-Q and Dominance voice personality ratings. Non-transformed scores are reported in pairwise correlations. Relations between the Dominant and Valence voice personality ratings, RLSHS-A, paranoid thinking and imagery abilities were assessed with hierarchical regression analysis.

### Results

#### Relations between auditory hallucination-proneness (RLSHS-A), Valence and Dominance voice personality scores, paranoid thinking (PADS) and imagery abilities (Psi-Q)

Table 4 shows the correlation matrix between auditory hallucination-proneness scores (RLSHS-A), Valence and Dominance voice personality scores, paranoid thinking (PADS) and imagery abilities (Psi-Q). Auditory hallucination-proneness positively correlated with paranoid thinking scores (*p <* .001), imagery ability scores (*p* = .05) and negatively correlated with Dominance voice personality scores (*p* < .001), suggesting that the higher the hallucination-proneness score was, the higher the ratings of voices as dominant. Valence voice personality ratings positively correlated with Dominance ratings (*p =* .001).

**Table 4:** Correlations between RLSHS-A, positive and dominant voice personality scores, paranoid thinking (PADS) and imagery abilities (Psi-Q).

	Valence	Dominance	Paranoid Thinking	Imagery
RLSHS-A	-.01	-.33**	.61**	.22*
Valence	-	.63**	.01	-.16
Dominance	-	-	-.13	-.06
Paranoid Thinking	-	-	-	.04

* p < 0.05

**p < 0.001

#### Predicting hallucination-proneness (RLSHS-A) controlling for paranoid thinking (PADS) and imagery abilities (Psi-Q)

A hierarchical regression was performed to assess contributions of Valence and Dominance voice personality ratings, paranoid thinking (PADS) and imagery abilities (Psi-Q) in predicting auditory hallucination-proneness (RLSHS-A). Valence and Dominance voice personality ratings were entered in the first block, and paranoid thinking and imagery abilities scores in the second block, with auditory hallucination-proneness (RLSHS-A) as a dependent variable. Measures of multicollinearity were in the acceptable range (VIF<5). Both Block 1 (*F*(2, 76) = 4.11, *p* = .02) and Block 2 (*F*(4, 74) = 12.81, *p* < .001) significantly predicted auditory hallucination-proneness. In Block 1 (*R*^*2*^ *=* 0.097), only Dominance ratings predicted auditory hallucination-proneness (β = -.40, *p* = .007). The addition of paranoid thinking and imagery abilities in Block 2 made a significant change to the model (*R*^*2*^ *=* .41, Δ *R*^*2*^ = .31, Δ *F* (2,74) = 19.52, *p* < .001), where Dominant voice personality ratings, β = -.39, *p =* .002, paranoid thinking, β = .51, *p* < .001 and imagery abilities, β = .22, *p* = .02, significantly predicted auditory hallucination-proneness.

#### Predicting Valence and Dominance ratings controlling for voice gender

Two multiple regression analyses were carried out. The first analysis assessed the contribution of Dominance and Valence, with scores calculated separately for male and female speakers in these two dimensions, in predicting hallucination-proneness for male participants (RLSHS-A). The same analysis was repeated for female participants.

In the female sample, Dominance and Valence ratings of female and male speakers were used as the predictor variables (in total four predictors: Valence-Male, Valence-Female, Dominance-Male, Dominance-Female), while hallucination-proneness (RLSHS-A) was the outcome variable. Measures of multicollinearity were in acceptable range (VIF<5). The model significantly predicted hallucination-proneness, *R*^*2*^ = .30; *F*(4,36) = 3.86, *p* = .01, where Dominant female voice ratings were the only significant predictor of hallucination-proneness, β = -.46, *p* = .02. For the analysis in male participants, the overall model was not significant, *R*^*2*^ = .14, *F*(4,55) = 2.30, *p =* .07, but dominant male voice ratings were a significant predictor of hallucination-proneness, β = -.59, *p =* .006. Full analysis is available in Supplementary Materials (S1 Table).

#### Summary

The study replicated the procedure of Study 1 and found that ratings of voice Dominance were a significant predictor of auditory hallucination-proneness. It was further revealed that this was specific to the gender of the participants, i.e. Dominance ratings of female voices predicted auditory hallucination-proneness only in female participants, and dominance ratings of male voices predicted hallucination-proneness only in male participants. The addiction of paranoid thinking and imagery abilities scores made a significant change to the model, where all three predictors showed a significant relationship with auditory hallucination-proneness.

## Discussion

The aim of this study was to investigate the role of voice personality ratings in predicting auditory hallucination-proneness in two general population samples. This aspect of voice perception has not been previously studied in the context of hallucination-proneness. We tested this relation in two studies, looking at ratings of male and female voices on four personality characteristics related to the so-called ‘social voice space’ [10]–Dominance (aggressiveness and confidence) and Valence (trustworthiness and warmth). In Study 1 this was studied in a university sample, where paranoid thinking scores were the only significant predictor of hallucination-proneness. While we found that Dominance ratings of voices were a significant predictor of auditory hallucination-proneness, this was not significant after the inclusion of paranoia scores, and no significant relation between gender of participants and ratings of male and female voices was found either. Because of the high proportion of female student participants in Study 1, we tested the same procedure in a more representative sample, also recruited online. In Study 2, we found that Dominance but not Valence voice personality ratings significantly predicted auditory hallucination-proneness. Interestingly, it was also found that this relation was gender-specific, i.e., dominance ratings of female voices predicted auditory hallucination-proneness in female participants only, while dominance ratings of male voices were associated with hallucination-proneness scores in male participants only (although this model was not significant). Paranoid thinking and imagery abilities scores were also found to be a significant predictor of auditory hallucination-proneness in Study 2.

The results of the study show that auditory hallucination-proneness is associated with the perception of voice personality, where increases in ratings of voice Dominance are associated with increase of hallucination-proneness scores. Research shows that, in the general population, there seems to be a consistency in recognising emotions communicated through voices [49, 50]. The research on voice perception also highlights an automatic aspect of voice perception where decisions about voices are processed in milliseconds [4]. Although the present study did not apply any measures to indicate rapid cortical processing of voices, the results show that minimal exposure to a person’s voice (average 670ms) can suggest individual differences in how voices were rated, which might be associated with important traits, such as hallucination-proneness. This might suggest that vocal perception in people prone to hallucinations might be biased towards perception of dominant personality traits as more intense. Research on emotion recognition in schizophrenia has suggested that emotion recognition might be compromised in this group, when observing other people’s faces [51, 52] and when listening to strangers’ voices [30, 53]. This usually manifests in a diminished ability to recognise the emotional categories. Higher judgements of personality characteristics for people who scored higher on hallucination-proneness in this study could reflect a similar vocal processing pattern, where higher personality judgements show inaccuracy in recognition of personality features. This judgement pattern appears to be specific to negative/dominant features of personality only, as the Valence scores did not show any significant relationship with hallucination-proneness. It would be worthwhile to investigate the perception of personality features further across different perceptual modalities in the future studies. It is unclear if similar relation between hallucination-proneness and perception of personality might look like in different modalities.

Although the results are from non-clinical samples, they have significant implications for understanding of voice perception in psychopathology. It would be worthwhile to test this paradigm in a sample of non-clinical voice-hearers as a next step to finding more about the link between voice perception and hallucinations. One limitation of this study is that it relied on a self-report questionnaire to measure auditory hallucination-proneness, which did not allow for thorough assessment of individual mental health history and substance abuse. In Study 2, the sample was pre-screened for history of mental illness through the participant recruitment platform Prolific; however, no such measures were used in Study 1. Given that the present study demonstrated that auditory hallucination-proneness is associated with high ratings of dominant personality traits, we could predict that the pattern would be similar in a clinical sample as well. This might be important for understanding the relationships and attitudes to hallucinated voices. Understanding how voice hallucinations are perceived is important in potentially improving one’s relationship with one’s voices. Perceived agency and personality of hallucinated voices are significant components defining these experiences [22]. Studying the association between voice personality perception and hallucination-proneness in a sample of clinical and non-clinical voice-hearers would be worthwhile in exploring the perception of the hallucinated voices.

Interestingly, the association of voice dominance ratings and hallucination-proneness seemed to be gender-specific. Ratings of female voices as dominant only significantly predicted hallucination-proneness in females while, among male participants, only dominance ratings of male voices showed a non-significant trend towards predicting hallucination-proneness. These results seem to suggest a heightened sensitivity in processing of voices of one’s own gender. Research on vocal emotion recognition has previously demonstrated advantages in accuracy in recognition of emotion in females [50, 54], where females have been found to be the most accurate in listening to female vocalisations [50]. The opposite has been found for males, who show the lowest accuracy in vocal male recognition [50]. Recognition of own-gender faces shows the same pattern, where women are more accurate at identification of female faces, a bias that has not been found to be present in male subjects [55]. Previous studies on voice perception indicate differences in perception of female and male voices, with perceived female attractiveness ratings highly correlated with judgements of valence. On the other hand, high ratings of dominance and aggressiveness in males have been found to be indicators of judged male attractiveness [10]. Interestingly, in the psychopathology literature, hallucinated male voices tend to be perceived as more dominant in nature, and this is true for both male and female patients [43]. Male voices have been found to be more commonly reported in clinical voice-hearers while male and female voices are heard more equally by non-clinical voice-hearers [56, 23].

Because of the link between psychosis and paranoia, and because paranoia may influence perceived voice personality ratings, we tested whether paranoid thinking might be involved in the relation between voice personality ratings and hallucination-proneness. The results showed that paranoid thinking and hallucination-proneness were positively correlated. The link between schizophrenia, psychosis and paranoid thinking is well-established, and may as well be linked to hallucination-proneness in non-clinical populations [57]. It appears that high ratings of dominant personality characteristics were not dependent on paranoid thinking tendencies in Study 2, as the ratings were found to be a significant predictor of hallucination-proneness even after addition of paranoia scores to the model. Perception of dominant personality characteristics as more extreme might be related to reasoning biases, for example ‘jumping to conclusions’ bias. This reasoning style has been widely found in schizophrenia and psychosis patients [58, 59] as well as in non-clinical participants with high scores of hallucination-proneness [60]. This relation could potentially be a subject for further studies exploring voice-perception and hallucination-proneness.

The results also revealed that higher imagery abilities scores were related to higher scores of auditory hallucination-proneness in Study 2. Evidence on the relation of imagination and hallucinations is mixed, although several studies do support this association (e.g., [38, 39]). Imagery ability was assessed based on visual, sensory and auditory imagery modalities. Evidence suggests enhanced auditory imagery abilities when it occurs alongside imagination in different modalities [61]. The imagery of visual features associated with the voices in the present study, might have conceivably affected the perception of personality features in voices. Although plausible, the present study did not directly investigate this hypothesis. Future research should explore how imagery modality might relate to perception of personality features in voices.

In typical voice perception, is not clear how personal life experiences shape the perception of other peoples’ voices. It is plausible that negative associations originating from past relationships could make people more vigilant and prejudiced towards specific kinds of voices. People seem to be accurate at judging the physical characteristics of others from listening to their voices alone, which might be associated with the connection of voices and physical characteristics learnt through past relationships [8]—for example, by learning to associate more negative and dominant features with certain types of voices. In psychopathology, life experiences have been shown to be strongly associated with the content of hallucinated voices, particularly in relation to childhood trauma (for a review, see [62]). It has been observed that the hallucinated voices are directly related to documented personal experiences–often the voice takes form of the abuser’s voice [63]. Future studies should explore how this could be related to hallucination-proneness more broadly–for example how negative associations with voices from past relationships make people more paranoid and vigilant when they are perceived.

One limitation to consider when interpreting the results is that the voices used in the studies were not closely matched to the population tested. The voice actors recruited for creation of the stimuli came predominantly from the South East and Midlands areas of the UK. In Study 1, we recruited a sample of undergraduate students from across the UK and did not match their regional accents to those used in the task stimuli. The accent differences were also evident in Study 2, where many participants were from outside of the UK (nearly 64%), thus having different regional accents from the ones used in the study. Studies in voice perception have been more rigorous on that matter; for example, McAleer et al. [10] have used predominantly Scottish accents matched to participants’ own accents. It is not clear what effect the actors and participants’ accents might have on the results; however, the fact that the study showed a relation between voice personality judgements and hallucination-proneness could suggest the effect might generalise. Nevertheless, future studies should examine this relation systematically.

Another limitation of this study is that it was carried out online, thus eliminating the possibility of controlling the testing environment (e.g., ensuring it is being carried out in a quiet environment). We introduced checks throughout the questionnaires in the study to monitor participants’ attention; however, such checks were impossible to carry out in the voice personality task.

To summarise, the present study showed that ratings of voices on a Dominance personality dimension are significant predictors of auditory hallucination-proneness, where higher rating scores are associated with higher hallucination-proneness. Interestingly, further analysis revealed that this is specific to the gender of the participant and the actor making the articulations–only Dominance ratings of female voices predicted auditory hallucination-proneness in female participants, and only male Dominance ratings were significant predictors in male participants. We also found that paranoid thinking and imagery abilities are significant predictors of auditory hallucination-proneness. To our knowledge this is the first study exploring the relationship of perceived voice personality features and hallucination-proneness. These findings are of importance to psychopathology research as they can further our understanding of why people experience distressing AVH. Future research should investigate voice personality perception in both clinical and non-clinical voice-hearers to test whether a similar relationship is found in these groups, as well as its relation to hallucinated voices.

### Ethical standards

Ethical approval was given by Department of Psychology Ethics Sub-committee at Durham University. The study was conducted in accordance with the ethical standards laid down in the 1964 Declaration of Helsinki and its later amendments. All participants gave informed written consent prior to their inclusion in the study.

## Supporting information

S1 Table Associations between ratings of personality characteristics in male and female voices, and auditory hallucination-proneness, in both male and female participants. (DOCX)
