# Supplementary material for: Reading characters in voices: Ratings of personality characteristics from voices predict proneness to auditory verbal hallucinations
Source: PLoS One. 2019 Aug 12;14(8):e0221127. doi: 10.1371/journal.pone.0221127 (PMC6690516; doi:10.1371/journal.pone.0221127)
Supplement: S1 Table — (DOCX) [file pone.0221127.s001.docx]

S1 Table – Results of regression analyses investigating the associations between ratings of personality characteristics in male and female voices, and auditory hallucination-proneness, in both male and female participants.

|  | | Participants | | | | | | | | | | | |  | |
| --- | --- | --- | --- | --- | --- | --- | --- | --- | --- | --- | --- | --- | --- | --- | --- |
|  |  | Male | | | | | | Female | | | | | |  |  |
|  |  | Dominance | | | Valence | | | Dominance | | | Valence | | |  |  |
|  |  | B | **β** | *p* | B | **β** | *p* | B | **β** | *p* | B | **β** | ***P*** | |  |
| Voice |  |  |  |  |  |  |  |  |  |  |  |  |  | |  |
|  | Male | -.006 | -.59 | .006 | .003 | .23 | .24 | -.002 | -.16 | .46 | .000 | .009 | .96 | |  |
|  | Female | .002 | .18 | .38 | .001 | .06 | .73 | -.007 | -.46 | .02 | .001 | .09 | .60 | |  |
|  | *R^2^* | .14 | | | | | | .30 | | | | | | |  |
|  | *F* | 2.30, *p* = .07 | | | | | | 3.96, *p* = .01 | | | | | | |  |
